# Automated Detection of *P*. *falciparum* Using Machine Learning Algorithms with Quantitative Phase Images of Unstained Cells

**DOI:** 10.1371/journal.pone.0163045

**Published:** 2016-09-16

**Authors:** Han Sang Park, Matthew T. Rinehart, Katelyn A. Walzer, Jen-Tsan Ashley Chi, Adam Wax

**Affiliations:** 1 Department of Biomedical Engineering, Duke University, Durham, North Carolina, United States of America; 2 Department of Molecular Genetics and Microbiology, Duke University, Durham, North Carolina, United States of America; 3 Duke Center for Genomic and Computational Biology, Duke University, Durham, North Carolina, United States of America; Johns Hopkins University Bloomberg School of Public Health, UNITED STATES

## Abstract

Malaria detection through microscopic examination of stained blood smears is a diagnostic challenge that heavily relies on the expertise of trained microscopists. This paper presents an automated analysis method for detection and staging of red blood cells infected by the malaria parasite *Plasmodium falciparum* at trophozoite or schizont stage. Unlike previous efforts in this area, this study uses quantitative phase images of unstained cells. Erythrocytes are automatically segmented using thresholds of optical phase and refocused to enable quantitative comparison of phase images. Refocused images are analyzed to extract 23 morphological descriptors based on the phase information. While all individual descriptors are highly statistically different between infected and uninfected cells, each descriptor does not enable separation of populations at a level satisfactory for clinical utility. To improve the diagnostic capacity, we applied various machine learning techniques, including linear discriminant classification (LDC), logistic regression (LR), and *k*-nearest neighbor classification (NNC), to formulate algorithms that combine all of the calculated physical parameters to distinguish cells more effectively. Results show that LDC provides the highest accuracy of up to 99.7% in detecting schizont stage infected cells compared to uninfected RBCs. NNC showed slightly better accuracy (99.5%) than either LDC (99.0%) or LR (99.1%) for discriminating late trophozoites from uninfected RBCs. However, for early trophozoites, LDC produced the best accuracy of 98%. Discrimination of infection stage was less accurate, producing high specificity (99.8%) but only 45.0%-66.8% sensitivity with early trophozoites most often mistaken for late trophozoite or schizont stage and late trophozoite and schizont stage most often confused for each other. Overall, this methodology points to a significant clinical potential of using quantitative phase imaging to detect and stage malaria infection without staining or expert analysis.

## Introduction

Malaria is a parasitic infectious disease caused by *Plasmodium* species, with *P*. *falciparum* being the most deadly and clinically relevant. This parasite has a complex intra-erythrocytic life cycle, moving through several stages of development while consuming the hemoglobin of the red blood cell (RBC). The gold standard for malaria diagnosis is manual microscopic evaluation of Giemsa stained blood smears. However, the utility of this approach is limited by the skill of an expert microscopist. Further, both the staining process and microscopic examination can be time consuming [1]. Therefore, there is an unmet need to bypass these requirements to allow easy detection and staging of malaria infection.

The aim of this manuscript is to report on the development of a method to automatically detect *P*. *falciparum* infection in unstained blood samples without human interpretation. Hänscheid *et al*. reported automated malaria diagnosis, using the Cell-Dyn full blood count analyzer that can distinguish abnormal monocytes and neutrophils containing birefringent hemozoin, showing 48.6% sensitivity and 96.2% specificity [2,3]. Several previous efforts have sought to use machine learning algorithms to detect malaria infection by automated analysis of microscopic images of stained red blood cells [4–6], achieving 84–95% accuracy in detecting parasites. These approaches can improve detection by removing the need for manual evaluation. However, they still rely on brightfield imaging of fixed and stained red blood cells. New imaging approaches can provide additional information that can potentially be used to improve automated detection. For example, recent studies using quantitative phase measurements have shown the ability to discern structural features indicative of *P*. *falciparum* infection in live, unstained blood cells [7]. Quantitative phase imaging (QPI) has been previously used to study morphological and temporal characteristics of individual cells *in vitro* by defining many metrics related to structural mechanics [8], molecular content [9], and dynamic responses to a wide range of stimuli with nanoscale sensitivity [10,11]. However, even with the wealth of information available through QPI, we still lack an automated algorithm that can discriminate malaria infection with sufficient accuracy to realize the clinical potential. Recent efforts from the group of B. Javidi have examined shape correlation of RBC images across several focal planes and achieved 86% discrimination accuracy [12]. Here, we seek to further improve the discrimination capacity of automated analysis by using multiparametric characterization of individual blood cells based on morphological descriptors extracted from quantitative phase images of live, unstained red blood cells. We have constructed machine learning algorithms using morphological descriptors of each cell extracted from quantitative phase images rather than the image data itself. Use of these parameters reduces the size of both training and test sets to allow the analysis of larger numbers of cells than previous studies using QPI. The resulting algorithms allow identification of malaria infection with high accuracy (>99%) and good discrimination of infection stage.

## Materials and Methods

### Ethics statement

This study was conducted with the approval of the Duke University Institutional Review Board (IRB), and the participant provided a written informed consent to participate in this study.

### Blood sample preparation

A whole blood sample was collected from a healthy, non-pregnant donor with informed written consent. In order to isolate red blood cells, purification protocols outlined by Sangokoya *et al*. were followed [13]. The fresh human blood sample was diluted in half by adding a volume of PBS equal to the blood volume. Then, the blood suspension was carefully layered on top of Ficoll, in an amount equal to the blood volume, in 50 cc conical tube. The cells were spun at 1500 rpm for 35 minutes at 25°C with no break. After the spin, RBC pellets at the bottom were isolated by removing the supernatant top layer, including white blood cells (WBC), and washed once in PBS.

RBCs were infected with *P*. *falciparum* strain, 3D7A, and synchronized using methods described by Saliba and Jacobs-Lorena [14]. During the 48-hour life cycle, infected RBCs were isolated from the general RBC population by magnetic sorting via a MACS magnet (Miltenyi Biotec) to separate uninfected RBCs from those containing parasites. Briefly, when most parasites are observed to be trophozoites or schizonts in a 30 mL culture, 5 mL of cultured cells are centrifuged at room temperature for 5 minutes at 1000 rpm (201 x g). Meanwhile, a prewarmed LS column (Miltenyi Biotec) is placed on a MACS magnet and is equilibrated with 5 mL of incomplete medium at 37°C. Supernatant from pelleted culture is removed, resuspended in 5 mL of incomplete medium and run through the LS column. The column is washed three times with 5 mL of incomplete medium at 37°C. The column is then carefully removed from the magnet, placed in a 15 mL conical tube, and eluted with 3 mL of incomplete medium at 37°C. The resuspended parasites are centrifuged at room temperature for 5 minutes at 1000 rpm (201 x g). The supernatant is removed, and the parasites are resuspended in 1 mL of PBS containing calcium chloride and magnesium chloride.

**Fig 1:**
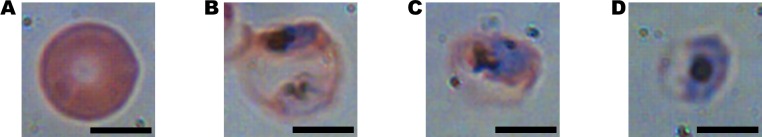
Bright-field microscopy images. Bright-field microscopy images: (A) uninfected RBC (B-D) RBCs with malaria parasite in early trophozoite, late trophozoite, and schizont stages respectively (scale bars = 5μm).

Parasite-infected red blood cells that were isolated using the magnetic sorting technique were imaged label-free in an aqueous environment (99:1 Dulbecco’s phosphate buffered saline, D8662 Sigma-Aldrich, to bovine albumin fraction V (7.5%), 15260–037 Gibco) using the QPS system at multiple time-points throughout the 48-hour life cycle: early trophozoite (24 hrs), late trophozoite (36 hrs), and schizont (48 hrs) stages (Distribution of RBC types, Table 1). Since RBCs with malaria parasites in ring stage, 12 hours post synchronized infection, could not be isolated with the magnetic sorting technique, they are not included in this study. Bright-field images of the histologic slides were made by fixing and staining RBCs with parasites at different stages of infection along with uninfected RBCs as shown in Fig 1.

**Table 1:** Distribution of RBC types used for imaging experiments.

Sample type	# of cells (N)	Time after infection [hours]
**Uninfected (S1 Fig)**	413	—
**Early trophozoite (S2 Fig)**	173	24
**Late trophozoite (S3 Fig)**	314	36
**Schizont (S4 Fig)**	337	48

White blood cells were also separated from the whole blood sample following the RBC isolation protocol, outlined above, until the centrifugation step. After spinning the cells, the upper layer containing plasma and platelets was removed without disturbing the WBC layer. WBC are carefully collected and washed in PBS + 10% FBS. The solution was then pelleted at 1500 rpm for 10 minutes and the supernatant was aspirated to isolate the WBCs. The isolated WBCs were washed and resuspended in PBS.

### Quantitative phase spectroscopy

**Fig 2:**
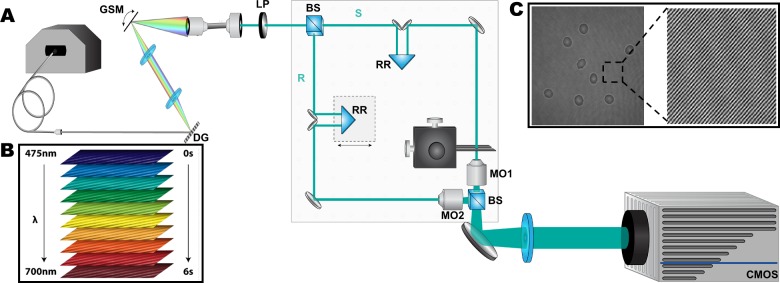
QPS system diagram. (A) Quantitative phase spectroscopy system: (DG) diffraction grating, (GSM) galvanometric scanning mirror, (LP) linear polarizer, (BS) beam splitter, (RR) retroreflectors, (MO) microscope objective. Path-matched sample (S) and reference (R) beams create off-axis interferograms imaged by a CMOS camera. (B) Interferogram spectral sweep from 475–700nm at 5nm increments in ~6s (C) Interferogram with corresponding fringes created by off-axis angle difference between the sample and reference arms.

We have used our quantitative phase spectroscopy system (QPS, Fig 2) [15] to image red blood cells. The digital holography system uses a supercontinuum laser source (Fianium SC-400-4) that is spectrally filtered to select a 1.12nm spectral full-width at half-maximum bandwidth from the broadband light with a variable center wavelength that is tuned across 475nm–700nm in 5 nm increments. This bandwidth is broad enough to reduce speckle from coherent artifacts but produces a reasonably long coherence length (ranges from 83–193 μm, depending on the center wavelength) such that the interferometric efficiency is not significantly degraded across the field of view.

The system employs a custom scheme to implement a rapidly-tunable spectral filter [15].The filter uses a 300 lp/mm transmission diffraction grating (DG) with a galvanometric scanning mirror (GSM) to couple the selected wavelengths into a single-mode fiber so that it may be introduced to the interferometer. The fiber output passes through a linear polarizer (LP) before entering the off-axis Mach-Zehnder interferometer as a collimated beam. In the interferometer, a beam splitter (BS) separates the illumination light into sample (S) and reference (R) arms that are path-matched within the coherence length of the filtered light using mirror-based retroreflectors (RR) on translation stages. The propagation angle of the reference arm is offset with respect to the sample arm to cause an angle difference between the two beams, creating off-axis interferograms (Fig 2C) that are detected by the CMOS camera (Photron FastCam SA-4, 1024×1024 px, 10-bit data capture). Matched microscope objectives, MO1 and MO2 (Zeiss Plan-NeoFLUAR 40× 0.75NA) are used in each arm, creating an image of the sample with an effective magnification of ~107x.

The interferograms are digitally processed, as described previously [15,16], to produce quantitative phase images, Δ*ϕ*(*x*,*y*). Processing steps include: (1) Fourier transforming the interferogram, (2) spatially filtering around the carrier frequency in the Fourier domain to isolate one of the complex conjugates, (3) re-centering the filtered two dimensional spatial frequency information and demodulating the complex wave, (3) inverse Fourier transforming the frequency information to produce a complex image of the wavefront differences containing both amplitude and phase information. A hyperspectral set of reference holograms of media-only fields of view (FOV) is captured separately and subtracted from the corresponding sample images of RBCs to remove phase artifacts due to non-uniformities in illumination. Any low-order background phase, variations caused by temporal drift between the two interferometer arms, are then removed by fitting the images to 5^th^-order polynomials. Changes in optical path length are calculated as:

ΔOPL(x,y,λ)=Δn(x,y,λ)∙h(x,y)=Δϕ(x,y,λ)∙λ/2π (1)

where *h*(*x*,*y*) is the height map of the object and Δ*n*(*x*,*y*,*λ*) is the refractive index map. Each data set comprises a distribution of optical path lengths across a range of several wavelengths. The set of optical path length maps are then averaged across the wavelengths to obtain an image with further reduced coherent noise artifacts [15]. Spatial noise of media-only background images is measured to be 7.5 mrad, corresponding to a Δ*OPL* sensitivity of 0.69 nm. Spectrally-averaged images are digitally refocused using a previously described algorithm [17] and red blood cells are automatically segmented from each FOV by applying joint optical path length and area thresholds. Within each FOV, all objects with Δ*OPL* > 10nm are identified as potential RBCs. Upper and lower area thresholds are used to exclude objects that are significantly bigger or smaller than known RBC size [18] such as cell clumps or fragments, free parasites, and non-RBC objects.

### Morphological parameters

In order to characterize the different types of RBC populations, 23 morphological metrics, listed in Table 2, are extracted from the isolated cells using both standard packages in MATLAB and customized algorithms.

**Table 2:** Morphological parameters used to describe RBCs.

Parameters	Definition
Max OPL	Δ*OPL*_*MAX*_(*x*,*y*)
Mean OPL	$\Delta \bar{OPL}(x,y)$
Median OPL	$\Delta \tilde{OPL}(x,y)$
Optical volume [17]	∫∫ΔOPL(x,y)dxdy
Minor axis length	Shortest line across a cell’s binary mask through its centroid
Major axis length	Longest line across a cell’s binary mask through its centroid
Perimeter	Outline of a cell’s binary mask
Area	Extent of a cell’s binary mask
Elongation	$\frac{Major\;axis\;length}{Minor\;axis\;length}$
Equivalent diameter	$2\sqrt{\frac{Cell\;area}{\pi }}$
Eccentricity	$\frac{Foci\;length}{Major\;axis\;length}\;(Foci\;length=2\sqrt{{\left(\frac{Major\;axis\;length}{2}\right)}^{2}-{\left(\frac{Minor\;axis\;length}{2}\right)}^{2}})$
Standard deviation OPL	$\sigma_{\Delta OPL}=\sqrt{\frac{\sum {\left(\Delta OPL(x,y)-\Delta \bar{OPL}(x,y)\right)}^{2}}{n}}$
Variance OPL	$\sigma_{\Delta OPL}^{2}$
Interquartile range OPL	Difference between the upper and lower quartiles that measures midspread of ΔOPL
Skewness	Asymmetry of ΔOPL’s histogram with respect to its mean
Kurtosis	Shape of peaks in ΔOPL’s histogram
Mean symmetry	Average rotational symmetry value
Min symmetry	Minimum rotational symmetry value
Maximum gradient	Maximum of ‖∇OPL(*x*,*y*)‖
Mean gradient	Average of ‖∇OPL(*x*,*y*)‖
Centroid vs. Center of mass	Physical skewness of a cell
Upper quartile mean OPL	Average of largest 25% ΔOPL values
Upper quartile mean gradient	Average of largest 25% ∇OPL values

**Fig 3:**
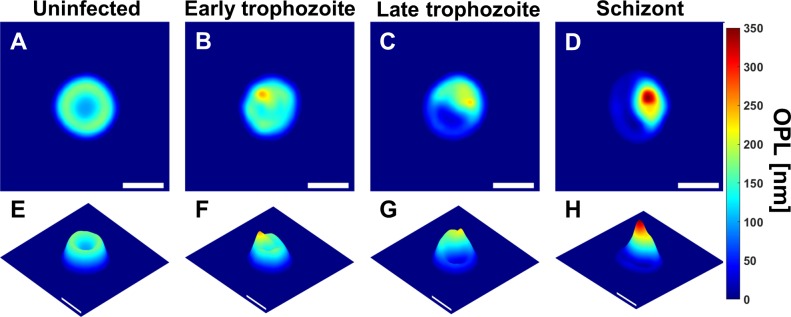
OPL maps. Uninfected RBC and RBCs infected by *P*. *falciparum* in early trophozoite, late trophozoite, and schizont stages represented respectively as: (A-D) OPL maps, (E-F) OPL maps from different viewpoint (scale bars = 5μm).

Quantitative measurements describing the geometric shape of the cells are calculated by analyzing the OPL maps (Fig 3A–3H) of the uninfected and malaria infected RBCs at various stages. Examples of geometric parameters used here are: 1) *Elongation*—the ratio of major axis length to minor axis length where major and minor axis are the longest and shortest lines across the centroid of an RBC’s binary mask, 2) *Equivalent diameter—*the diameter of a circle with the same area as that of the RBC, 3) *Eccentricity*—the ratio of the distance between the foci and the major axis length of a RBC describing the roundness of its shape.0020

**Fig 4:**
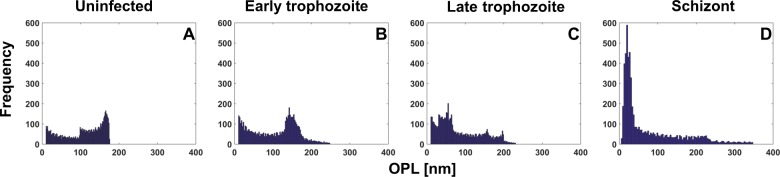
OPL histograms. (A-D) OPL histograms of uninfected RBC and RBCs infected by *P*. *falciparum* in early trophozoite, late trophozoite, and schizont stages respectively as shown in Fig 3.

In addition, statistical features based on the histograms of the OPL distribution for each cell (Fig 4A–4D) are also used to characterize the RBCs. Both skewness and kurtosis, also known as 3^rd^ and 4^th^ central moments respectively, describe the shape of the histogram: *skewness* represents asymmetry of data points around the mean value while *kurtosis* characterizes the heavy tails of a histogram that can be related to the shape of the peaks in the distribution. These parameters can be calculated as:

$$Skewness={E(\Delta OPL(x,y)-\Delta \bar{OPL}(x,y))}^{3}/{\sigma_{OPL}}^{3}$$ (2)

$$Kurtosis={E(\Delta OPL(x,y)-\Delta \bar{OPL}(x,y))}^{4}/{\sigma_{OPL}}^{4}$$ (3)

where *E*(*t*) is the expected value.

**Fig 5:**
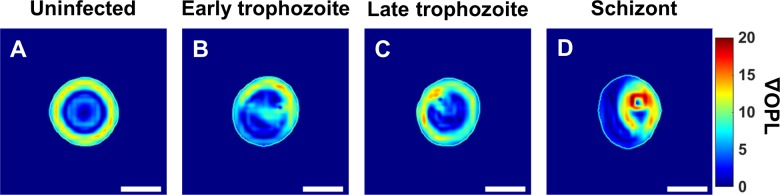
Gradient maps. (A-D) Gradient maps of uninfected RBC and RBCs infected by *P*. *falciparum* in early trophozoite, late trophozoite, and schizont stages respectively as shown in Fig 3 (scale bars = 5μm).

Further parameters can be obtained by calculating the *gradient* of each cell’s phase changes. The rate of change in the height of different types of RBCs, as represented by gradient maps (Fig 5A–5D), identifies sharp changes in the thickness of infected RBCs, which could arise due to parasite infection. The magnitude of the gradient can be calculated as:

$$\|\nabla OPL(x,y)\|=\sqrt{{\left(\frac{\partial OPL(x,y)}{\partial x}\right)}^{2}+{\left(\frac{\partial OPL(x,y)}{\partial y}\right)}^{2}}$$ (4)

The symmetry of each cell can also provide valuable discrimination. *Symmetry* is calculated as the dot product of a rotated image of the RBC and the original image across a range of angles up to a full rotation referenced to the scalar product of the original cell image.

$$Symmetry(\theta )=\frac{\left(\Delta OPL(x,y)∙\;\Delta OPL(x^{\prime },y^{\prime })\right)}{{\left\|\Delta OPL(x,y)\right\|}^{2}}\;where\;\left[\begin{array}{c} x^{\prime }\\ y^{\prime } \end{array}\right]=\left[\begin{array}{cc} \cos\theta & \sin\theta \\ -\sin\theta & \cos\theta \end{array}\right]\;\left[\begin{array}{c} x\\ y \end{array}\right]$$ (5)

**Fig 6:**
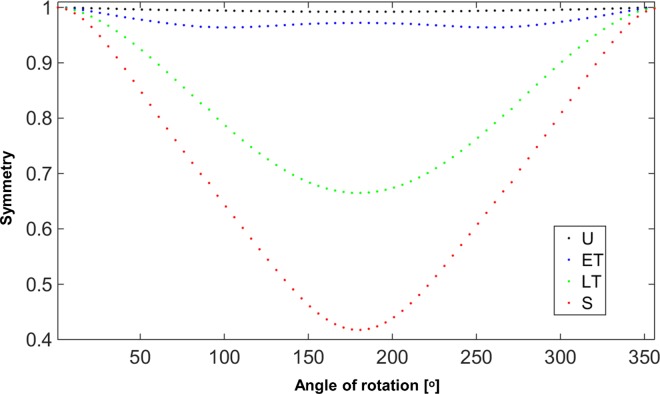
Symmetry values. Symmetry values of uninfected RBC and RBCs infected by *P*. *falciparum* in early trophozoite, late trophozoite, and schizont stages respectively as shown in Fig 3 versus angles of rotation.

Symmetry values for the uninfected RBC and the three different stages of parasite-infected RBCs shown in Fig 3 are plotted in Fig 6 across the range of rotation angles. Both mean and minimum symmetry values are used as descriptors of the red blood cells.

**Fig 7:**
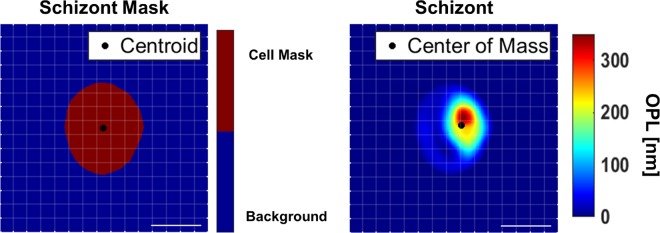
Centroid vs. Center of mass. Binary mask used to calculate centroid and ΔOPL map used to calculate center of mass for an RBC with *P*. *falciparum* at schizont stage (scale bars = 5μm).

Another way of representing asymmetry is by analysis of the differences between the centroid and center of mass. These both represent geometric centers of an RBC; however, centroid assumes uniform density across its area. For example, in Fig 7, the centroid of an RBC containing a parasite at schizont stage is calculated with a binary mask of the cell while the center of mass is calculated using the OPL map as a surrogate measure of mass distribution. Therefore, the difference between the two positions can be related to the magnitude of an RBC’s physical asymmetry.

Finally, the upper quartile of the OPL and OPL gradient histograms for each RBC are averaged to produce descriptors that reflect the differences in the thickest regions and greatest transitional regions, of cells, respectively. These metrics are expected to be directly related to the presence of parasites.

**Fig 8:**
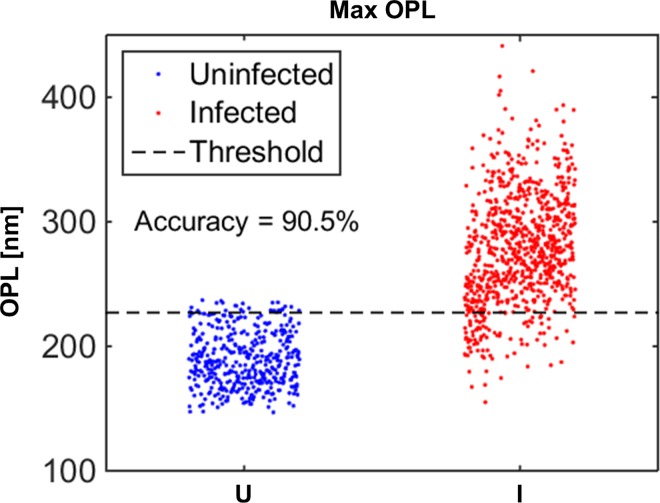
Population identification using maximum OPL. Comparison of maximum OPL for uninfected RBCs and RBCs with parasites at all of the stages combined. Optimal threshold for population identification based on maximum OPL results in 90.5% accuracy.

When the uninfected RBC population is compared against the malaria parasite-infected RBC population, all of the 23 morphological parameters listed in Table 2 show differences that are highly statistically significant when considered individually (P-value << 0.001). However, uninfected and infected RBC populations cannot be separated from each other when only one of these morphological parameters is used. For example, maximum optical path length, which produces the smallest p-value, can be used to determine a threshold of classification as shown in Fig 8. This parameter separates the two populations with 94.0% specificity, 88.8% sensitivity, and 90.5% accuracy. Since all of the 23 metrics measured by QPS result in highly statistically significant differences in describing the two different populations, machine learning systems are used to combine the parameters in a logical way to formulate algorithms that can better separate the populations.

### Machine learning algorithms

Machine learning systems build a predictive model based on identified inputs as a teaching or learning set and classify new datasets using a customized algorithm instead of following explicitly programmed instructions. In order to classify RBCs, 3 different types of machine learning techniques were examined based on their prediction accuracy and speed: linear discriminant classification (LDC), logistic regression (LR), and *k*-nearest neighbor classification (NNC). LDC, also known as Fisher discriminant classification, relies on linear combinations of training data that best separate different populations by finding a multidimensional axis that maximizes the between-population variability while minimizing the within-population variability. LR is an algorithm that determines a linear combination of parameters from training data based on the maximum likelihood method with logit link function. Unlike LDC and LR which create algorithms from the training data, NNC is an instance-based learner, which directly uses the training dataset for classification. When a new dataset is in need of classification, NNC finds *k*-number of closest points (*k* = 3) and classifies the new data according to the majority identification of those nearest neighbors. For our experiment, the *k* parameter has been determined by testing NNC’s performance using different *k* ranging from 2–5. These algorithms are used to make binary classification between uninfected cells and cells with malaria parasites in different erythrocytic stages. Also, their performances for determining the different stages of infection are evaluated through multinomial classification.

The predictive power of the supervised learning methods is assessed using *k*-fold cross-validation (*k* = 10). In order to validate a machine learning model, the dataset (N = 1237) which includes both infected and uninfected cells is randomly partitioned into 10 subsets that are roughly equal in size. Then, 9 of the subsets are used as the training dataset to create a model and the remaining subset is used as the testing set to measure its performance. Analysis with a different testing set is repeated until all 10 subsets have been used once as a testing set. To minimize variability, average performance of a model using 100 rounds of cross-validation with new subsets which are randomly partitioned each time is reported.

## Results

### Uninfected vs infected RBC

Machine learning algorithms are used to distinguish uninfected RBCs from 3 different hemozoin containing stages of *P*.*falciparum* infected RBCs (early trophozoite–ET, late trophozoite–LT, schizont–S). The performance of the three supervised learning methods, as evaluated using the 10-fold cross-validation technique, is summarized in Table 3.

**Table 3:** Performance of machine learning algorithms: Uninfected vs Infection stage.

	NNC			LR			LDC		
Stage (# of cells)	ET (173)	LT (314)	S (337)	ET (173)	LT (314)	S (337)	ET (173)	LT (314)	S (337)
**Sensitivity [%]**	**87.8** ± 0.7	**99.2** ± 0.3	**99.0** ± 0.2	**90.8** ± 0.4	**98.9** ± 0.2	**99.4** ± 0.2	**93.5** ± 0.3	**97.7** ± 0.3	**99.4** ± 0.04
**Specificity [%]**	**99.2** ± 0.2	**99.8** ± 0.01	**100**	**98.4** ± 0.2	**99.3** ± 0.1	**99.7** ± 0.1	**99.9** ± 0.1	**100**	**100**
**Accuracy [%]**	**95.8** ± 0.3	**99.5** ± 0.1	**99.6** ± 0.09	**96.1** ± 0.2	**99.1** ± 0.1	**99.6** ± 0.1	**98.0** ± 0.1	**99.0** ± 0.1	**99.7** ± 0.02

**Fig 9:**
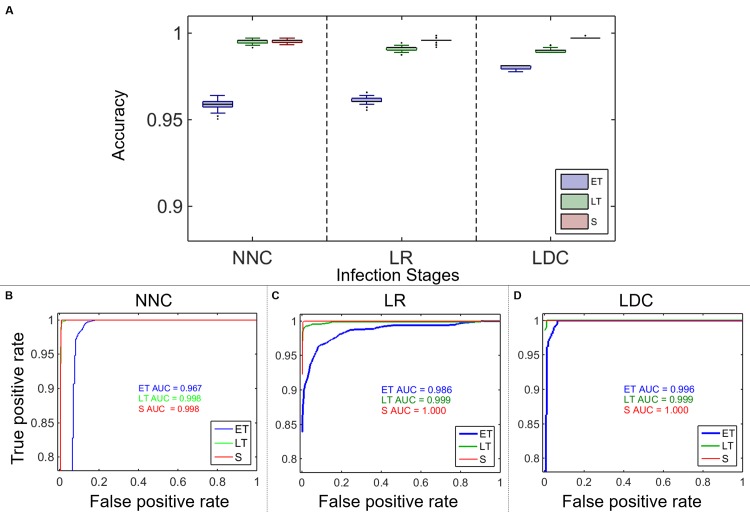
Uninfected vs. Infected RBC. A) Accuracy of nearest neighbor classification (NNC), logistic regression (LR), and linear discriminant classification (LDC) used to distinguish uninfected RBCs from RBCs infected with *P*.*falciparum* parasites in early trophozoite (ET), late trophozoite (LT), and schizont (S) stages. B- D) ROC curves and their corresponding AUC for NNC, LR, and LDC respectively.

All of the classification methods have higher specificities compared to their sensitivities when distinguishing uninfected from infected RBCs for all 3 stages of infection. The specificities ranged from 98.4% for LR with the early stage of infection (ET) to 100% for the best performing method (LDC) for both LT and S stages. Among all three methods, the worst performance was in distinguishing ET, with NNC offering the lowest sensitivity for this stage (87.8%) while that of LDC and LR methods were 93.5% and 90.8%, respectively. The overall accuracy of the classification methods are compared graphically in Fig 9A. Note that the accuracy remains over 95% for all of the stages and machine learning methods examined here. ROC curves with corresponding AUC values are shown in Fig 9B–9D.

All of the methods show very high accuracies, especially when they are applied to classify uninfected RBCs from RBCs with *P*.*falciparum* parasites in later stages: they are able to classify RBCs with *P*.*falciparum* parasites in schizont stage at or above 99.6% accuracies. These results are supported by the perfect and near-perfect AUC values in Fig 9B–9D. As expected, all of the machine learning algorithms show lower accuracies when differentiating RBCs with ET parasites because this early stage of infection exhibits less morphological changes.

The clinical utility of these approaches can be illustrated by calculating the positive and negative predictive values (PPV & NPV, Table 4). For cells with parasites in LT and S stages, the PPVs are both 100% using LDC. The perfect positive predictive values and specificities indicate that the classifier did not have any false positive outcomes where uninfected RBCs would be incorrectly classified as RBCs with parasites in either LT or S stages. The NPV values for all of the stages are above 95% indicating that there are only a few false negatives where infected RBCs are classified to be uninfected, mostly for early trophozoite stage. The NPV show errors ranging from 0.5% to 4.8% depending on infection stage (NNC: 8, 1, 2 / LR: 7, 3, 2 / LDC: 5, 6, 2 misclassified cells respectively for ET, LT, and S).

**Table 4:** Positive and negative predictive values.

	NNC			LR			LDC		
Stage (# of cells)	ET (173)	LT (314)	S (337)	ET (173)	LT (314)	S (337)	ET (173)	LT (314)	S (337)
**PPV [%]**	**97.8** ± 0.6	**99.7** ± 0.03	**100**	**95.9** ± 0.6	**99.1** ± 0.2	**99.7** ± 0.2	**99.8** ± 0.3	**100**	**100**
**NPV [%]**	**95.2** ± 0.3	**99.4** ± 0.2	**99.2** ± 0.2	**96.3** ± 0.2	**99.2** ± 0.2	**99.5** ± 0.1	**97.3** ± 0.1	**98.3** ± 0.2	**99.5** ± 0.06

### Discrimination of infection stages

**Fig 10:**
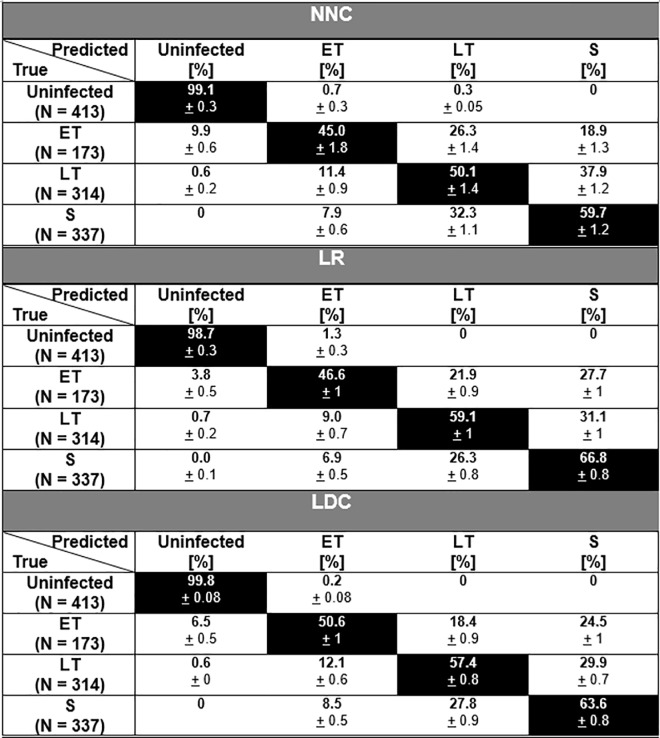
Infection staging. Table showing performance of multinomial machine learning algorithms: Infection stages.

**Fig 11:**
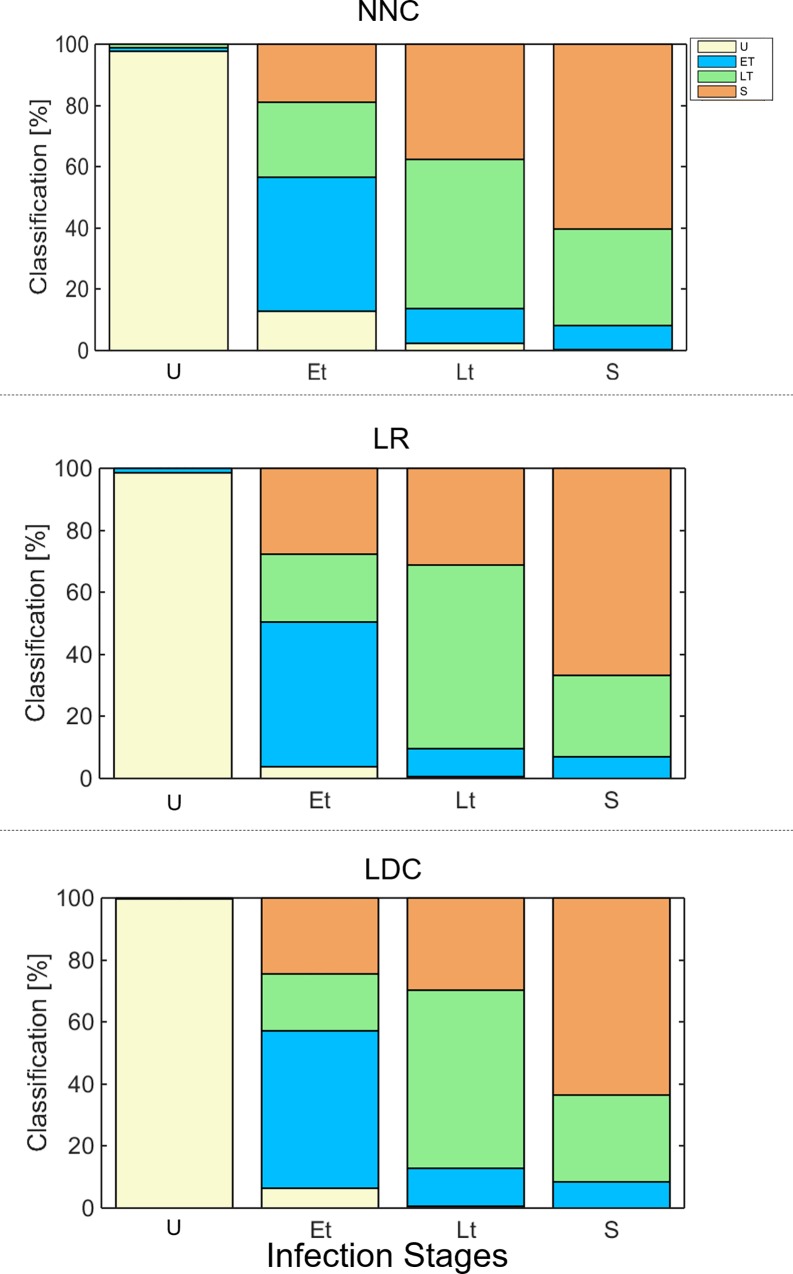
Stacked bar plot–infection staging. Performance of multinomial machine learning algorithms: Infection stages.

The results of multinomial classifications using the supervised learning algorithms, NNC, LR, and LDC, are shown in Fig 10. The classification of the cells, as determined by time after synchronized infection and confirmed with histological analysis, are listed in the left column and the percentages of predicted identities using the supervised learning systems are listed along the rows of the table. The highlighted diagonal table elements indicate the correct classifications. Fig 11 presents these classification results graphically in stacked bar plots.

As can be seen in Fig 10, NNC, LR, and LDC all have high specificities, the rate of true uninfected cells classified as uninfected, of 99.1%, 98.7%, and 99.8% respectively. Furthermore, none of the uninfected RBCs are classified as RBCs with schizont stage parasites and very few (1–7 cells) are classified as ET or LT. The majority of the cells across the different stages of infection are identified correctly using all three algorithms (NNC: 45.0%, 50.1%, 59.7% / LR: 46.6%, 59.1%, 66.8% / LDC: 50.6%, 57.4%, 63.6% respectively for ET, LT, and S). The performances of the classification algorithms for discriminating the various infection stages from each other are lower than the specificities of the multinomial classifications. However, the classification errors rarely confuse an infected cell for an uninfected one. 9.9% (~17 cells), 3.8% (~7 cells), 6.5% (~12 cells) of the total ET population are classified to be uninfected RBCs by NNC, LR, and LDC respectively. This type of misclassification is even lower for RBCs with parasites in LT and S stages for the algorithms, with the error rate dropping to 0.6% (2 cells) for LT and zero for S stage cells using both the NNC and LDC algorithm.

### Discrimination of white blood cells

**Fig 12:**
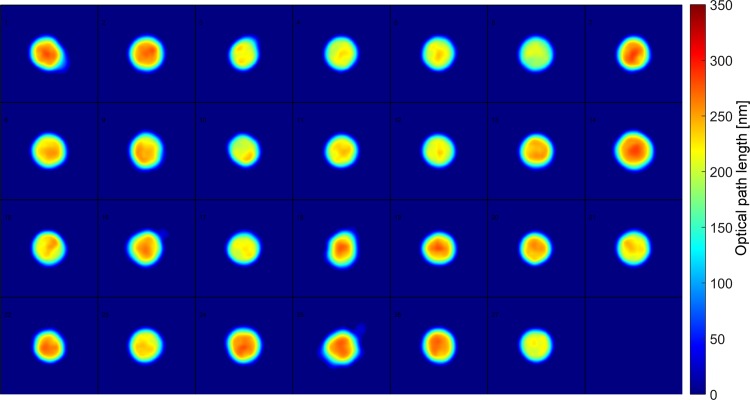
WBCs. WBCs, N = 27 (square tile = 20μm x 20μm).

In a further demonstration of the capabilities of this approach, white blood cells are separated from the red blood cells by fractionating whole blood and are imaged by QPS. Fig 12 below shows the OPL maps of fractionated WBCs.

The machine learning algorithms from the previous experiments, including multinomial NNC, LR, and LDC, that are trained with the uninfected and parasite-infected RBCs are used to classify WBCs in Fig 12. NNC and LDC predicted 24/27 WBCs (89%) and 19/27 WBCs (70%), respectively, to be uninfected RBCs and the rest to be RBCs with parasites in the early trophozoite stage while LR classified 9/27 cells (33%) to be uninfected RBCs and the other cells as RBCs with parasites in ET stage. Since our algorithms classify some of the WBCs as cells infected with malaria parasites, our system is limited to RBC samples that are isolated by whole blood fractionation.

## Discussion

Malaria infection is a leading cause of death worldwide that can be managed with early detection and proper treatment using artemisinin-based combination therapies [19]. The parasitemia percentage at which patients display symptoms of infection can range anywhere from 0.0002% to 0.7% depending on the severity of the infection and the level of immunity towards malaria parasites [20]. Peripheral blood smear screening using the light microscope can be very sensitive with the ability to detect malaria parasite densities as low as ~0.0001%. However, the accuracy of the technique is reduced for low-density parasitemia. Errors of identification have been previously reported for samples with parasitemia densities below 0.4% [21]. Also, microscopic examination of stained blood smears depends upon the expertise of trained microscopists and, therefore, is subject to humanistic error and variability. In regions where malaria is not endemic and malaria microscopy examination is not routinely performed, the sensitivity of malaria detection decreases significantly. A recent study in U.S. acute care settings showed 88% sensitivity in distinguishing patients infected with *P*. *falciparum* [22] and an earlier study in Canada reported that 59% of malaria cases were misdiagnosed [23]. In addition, manual diagnosis procedures are time consuming and labor intensive. This aspect is especially problematic since the majority of malaria-related deaths occur in low resource settings where the needed expertise is not easily found [24]. Therefore, quantitative assessment of malaria infection using automated methods can reduce the need for trained microscopists and assist clinicians to make better, faster decisions regarding malaria diagnosis.

Previously, Hänscheid *et al*. showed that a full blood count analyzer can be implemented as an automated malaria diagnosis tool using the depolarizing characteristic of hemozoin [2,3]. Although erythrocytes can produce depolarization when illuminated by laser light, monocytes and neutrophils do not unless they contain hemozoin, a birefringent byproduct of malaria parasites. The full blood count analyzer can detect malaria by measuring changes in the intensity of depolarized scattered light from WBCs, effectively detecting those with hemozoin. Although this approach showed specificity as high as 96.2%, the sensitivity was much lower at 48.6%. Also, hemozoin-containing monocytes have been found 2–3 weeks after the patients were parasitologically cured which may result in false positives after the treatment.

QPI has also been used to analyze RBCs infected by *P*. *falciparum* by characterizing their physical properties such as RBC volumes and shape correlation [7,12]. Kim *et al*. reconstructed 3-D optical refractive index (RI) tomograms of RBCs with malaria parasites at different stages of infection that were used to quantify various features such as cytoplasmic and parasite volumes. While this approach produces highly detailed maps of RI, the computation time is extensive. Further, although the examined parameters offer a useful characterization of cell changes due to infection, they do not appear to provide a suitable method for discrimination. Anand *et* al. used correlation coefficients based on the thickness distribution of RBCs at multiple reconstructed axial planes to separate RBC populations. This approach produced reasonable accuracy but did not provide sufficient discrimination to point to clinical utility. Computation times were not given but may be a barrier to examination of large numbers of cells with this approach.

In this work, we have used morphological parameters extracted from phase images of RBCs to build machine learning algorithms that show great performance in distinguishing uninfected vs. infected RBCs. One improvement is reducing the total processing time needed to evaluate a sample. After obtaining raw images from unstained blood samples, we can extract all of the relevant morphological features of the RBCs in a FOV (~10 cells) in less than 150 seconds which is much faster than previous efforts (15 sec/cell vs. 3000 sec/cell for Kim et al [7]). All data analysis was executed with custom scripts in MATLAB on a desktop computer (Intel Core i5 2400 CPU, 3.10GHz, 32 GB RAM). For clinical use, general machine learning algorithms, such as LDC and LR, can be created ahead of time with training data of known samples. Since it is not necessary to reconstruct new algorithms for each test sample, the analysis procedures can be accomplished within a short time. Also, population identification using extracted features and pre-built machine learning algorithms takes ~5ms for all of the cells in a FOV, which allowed us to characterize relatively large populations of different types of RBCs. Although the overall computation time is not yet clinically feasible, the approach can be further developed to enable higher throughput evaluation. Use of the parallel computing capabilities of a graphics processing unit (GPU) in addition to optimization of morphology extraction scripts could significantly reduce this computation time and will be an area of future work in this development.

As shown in Table 3, all of the machine learning algorithms identify uninfected and infected RBCs with high accuracies. They have higher specificities for all of the stages of infection indicating that they discriminate uninfected RBCs more effectively. Also, high PPV values in Table 4, which indicate low false positive outcomes, show the system’s potential application as a screening tool to exclude blood samples that do not require further examination by expert microscopists therefore expediting the total diagnostic process. Although the classifiers performed with lower NPV values indicating that some infected cells were incorrectly identified in this study, these rates are comparable to those obtained by trained microscopists [25].

Typically, malaria diagnostic modalities are compared to one another by the lowest detectable parasitemia percentages. Currently, the ability to evaluate our technique is limited by the total number of uninfected cells (413) that were imaged with the system. Due to the sample size, our technique cannot show diagnostic performance with samples that have parasitemia percentages below 0.2%. Further work with QPI and machine learning algorithms will seek to define their accuracy in determining parasitemia percentages in samples with controlled levels of infection that match the levels of the typical patients by increasing the sample size and creating a synthetic population of uninfected cell data based on random samples of the distribution of the 23 morphological parameters. Also, the ring stage, the earliest stage of the parasites that would complete the erythrocytic cycle, will be explored in the future which could require the use of additional parameters as input to the machine learning algorithms.

Currently, our system is limited to classifying red blood cells that have been separated using a whole blood fractionation method such as the one described in the blood preparation section. Confounding cells in whole blood samples, such as white blood cells and reticulocytes, each representing ~1% of the total number of blood cells, are likely to be misclassified by our algorithms which have been trained only with uninfected and parasite-infected RBCs. The classification results of the additional experiment with WBCs in Fig 12 show that current algorithms would only be useful if samples were prepared for analysis so that only RBCs were imaged by the QPS system. Also, patients with hemoglobinopathies and auto-hemolytic anemia, such as spherocytosis, will have RBCs that have different morphology compared to RBCs that were used to train our algorithms. These patients would require development of new algorithms which are trained with control groups that are more relevant to their conditions. In the future, we will conduct a more complete analysis of whole blood samples by training our algorithms on samples which include these confounding cells in order to make our system more clinically applicable.

Classification of different erythrocytic stages of malaria parasites can help choose treatment based on stage-specific sensitivity of antimalarial drugs [26–28]. Although the algorithm based classification of infection stages does not perform as well as binary classification of uninfected vs. infected RBCs, the multinomial classification still has high specificity and sensitivity where the vast majority of the cells are accurately classified according to the infection timeline. Also, it should be noted that the multinomial classifications maintain high performances when the cells with parasites are grouped across the different stages of infection with sensitivities as high as 97.7%, 98.9% and 98.4% respectively for NNC, LR, and LDA. While higher performance would be needed to rely on the automated algorithm for selecting treatment courses, the ability of the approach to detect infection suggests it can be used as a screening tool with further stage discrimination conducted via manual interpretation, if warranted.

## Conclusion

In our study, QPS is used to image RBCs infected by different stages of *P*.*falciparum* to distinguish them from uninfected RBCs. The physical descriptors of each population, extracted from the phase images, are used to train machine learning algorithms that classify RBCs with great accuracies. One of the main strengths of using machine learning algorithms to analyze the extracted parameters is that the identification of RBC infection will be based on quantified metrics and pre-built classifiers that requires minimal operator training. In order to enable automated imaging in the future, a microfluidic device with controlled flow rates can be combined with the analysis approach that would allow high throughput. This would permit rapid analysis of a blood sample at the point of care to assist the clinical decision of physicians. The World Health Organization has recommended a minimal standard of 95% sensitivity and specificity for diagnostic tools to be clinically useful when evaluating patients infected with *P*. *falciparum* densities of 0.0002% [29]. In the future, experiments involving samples that match parasitemia levels of typical malaria patients will be evaluated using our combined imaging and analysis modality. Currently, the supervised learning models are created exclusively with morphological parameters but further studies can be conducted to extract more information from additional cell properties, such as spectral features, to strengthen performance in distinguishing parasite-infected cells as well as their infection stages.

## Supporting Information

S1 Fig Uninfected RBCs. Uninfected RBCs, N = 413 (square tile = 20μm x 20μm).
(TIF)

S2 Fig RBCs infected with *P*.*falciparum* in early trophozoite stage. RBCs infected with *P*.*falciparum* in early trophozoite stage, N = 173 (square tile = 20μm x 20μm).
(TIF)

S3 Fig RBCs infected with *P*.*falciparum* in late trophozoite stage. RBCs infected with *P*.*falciparum* in late trophozoite stage, N = 314 (square tile = 20μm x 20μm).
(TIF)

S4 Fig RBCs infected with *P*.*falciparum* in schizont stage. RBCs infected with *P*.*falciparum* in schizont stage, N = 337 (square tile = 20μm x 20μm).
(TIF)

S5 Fig Overall pipeline. Pipeline showing the overall procedure.
(TIF)

S1 Table Cell properties. 23 morphological parameters for all of the RBCs.
(XLSX)
