# Supplementary figures and images for: Automated Detection of P. falciparum Using Machine Learning Algorithms with Quantitative Phase Images of Unstained Cells
Source: PLoS One. 2016 Sep 16;11(9):e0163045. doi: 10.1371/journal.pone.0163045 (PMC5026369; doi:10.1371/journal.pone.0163045)

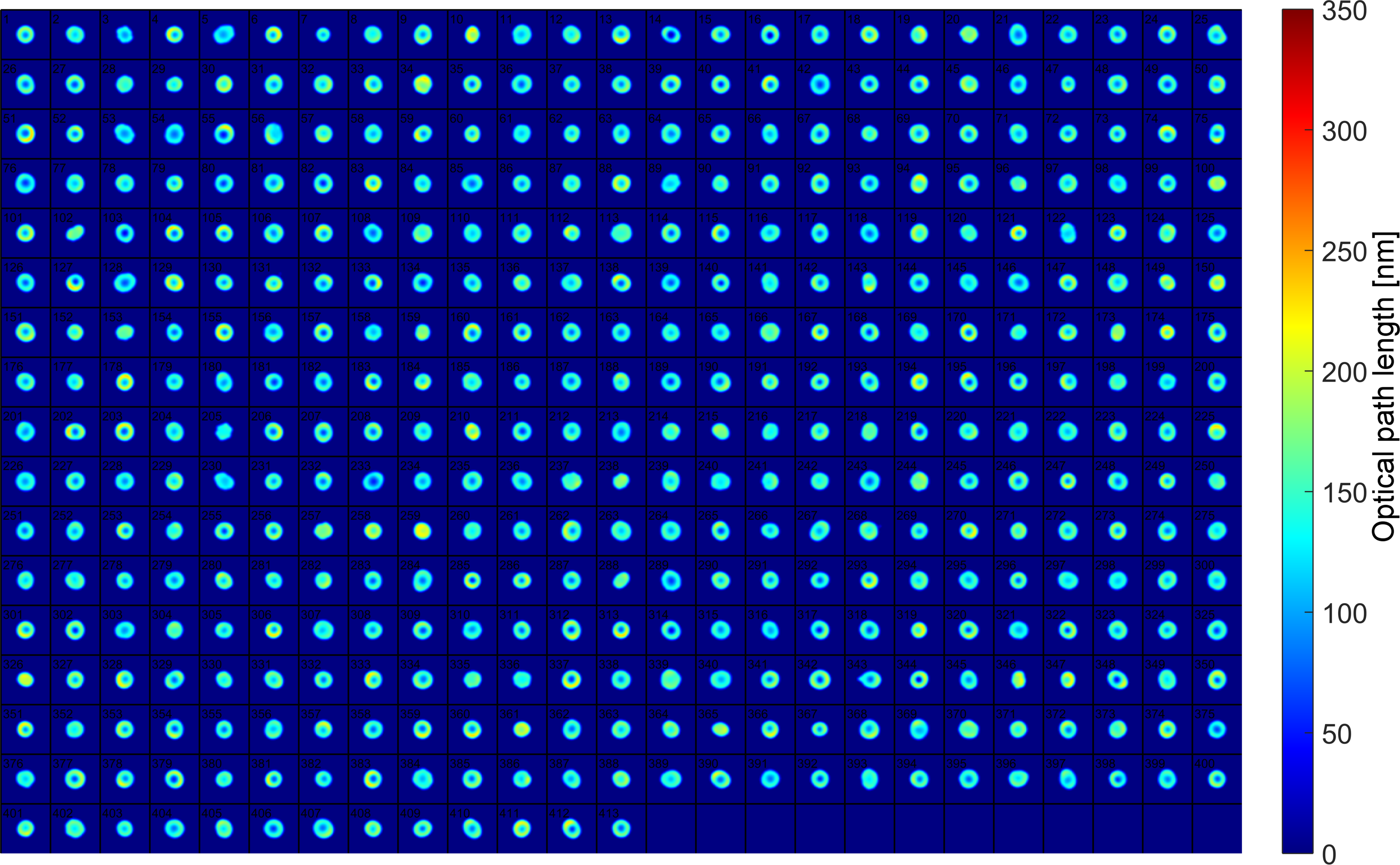

Supplement: S1 Fig — Uninfected RBCs, N = 413 (square tile = 20μm x 20μm). (TIF) [file pone.0163045.s001.tif]

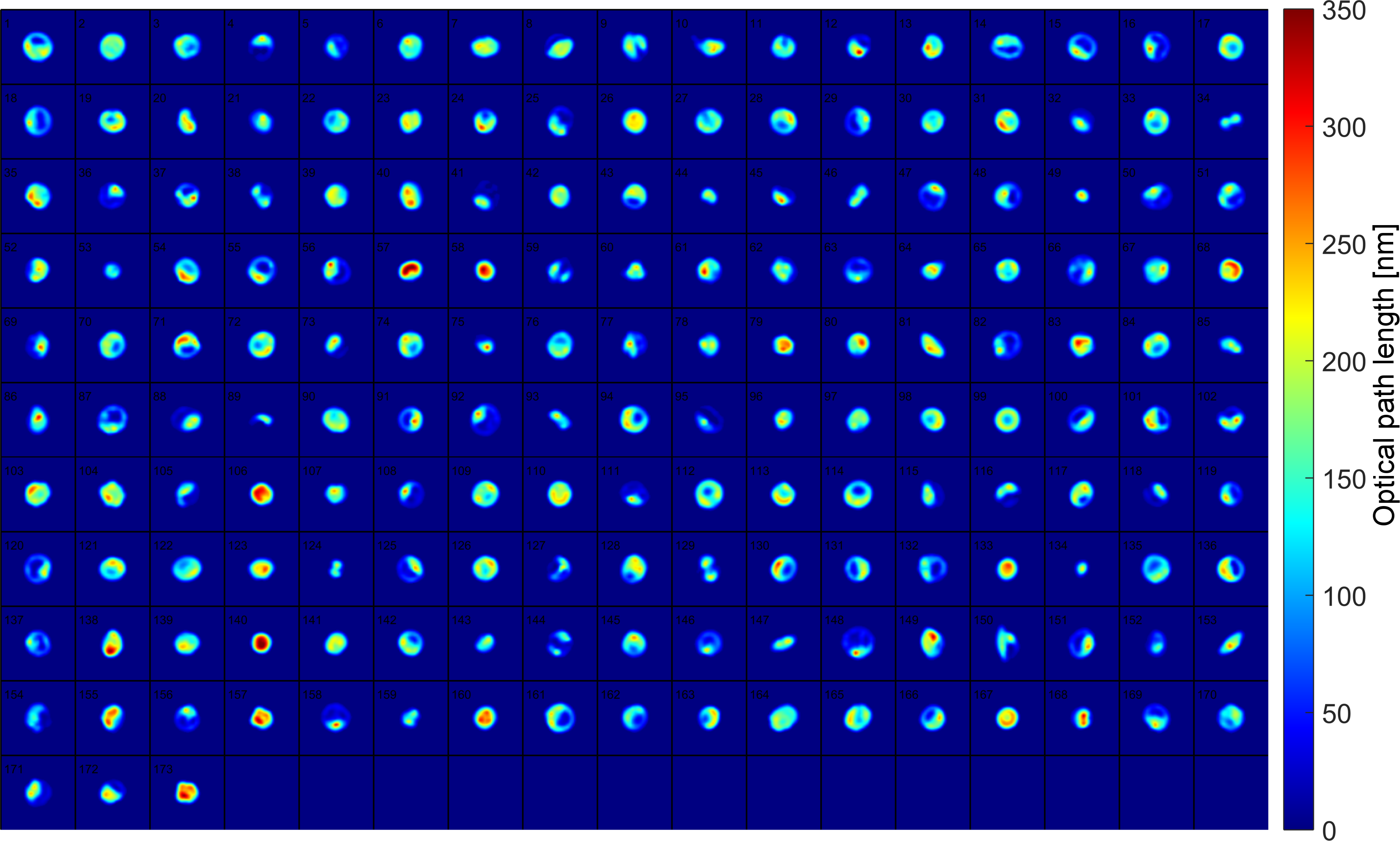

Supplement: S2 Fig — RBCs infected with P.falciparum in early trophozoite stage, N = 173 (square tile = 20μm x 20μm). (TIF) [file pone.0163045.s002.tif]

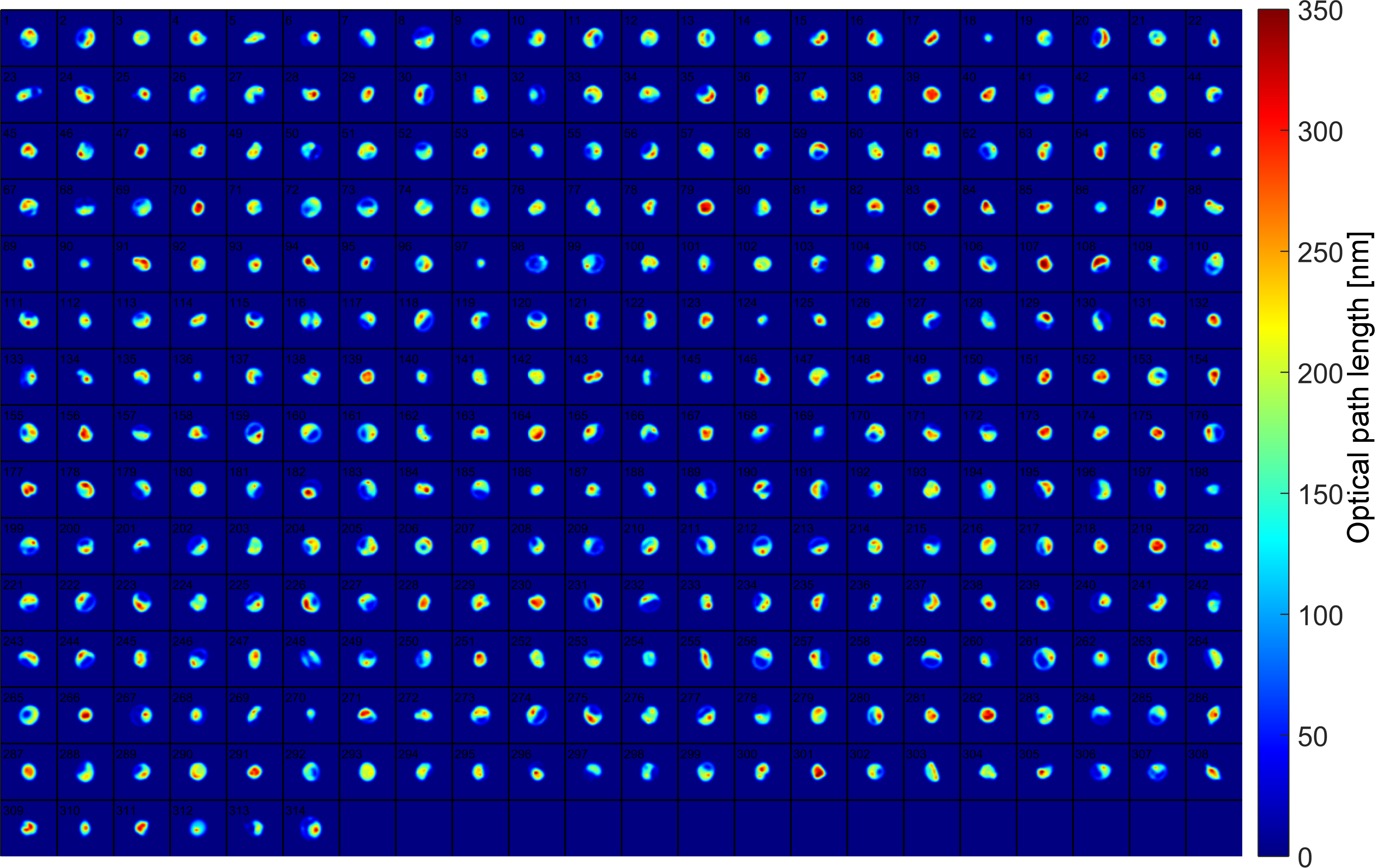

Supplement: S3 Fig — RBCs infected with P.falciparum in late trophozoite stage, N = 314 (square tile = 20μm x 20μm). (TIF) [file pone.0163045.s003.tif]

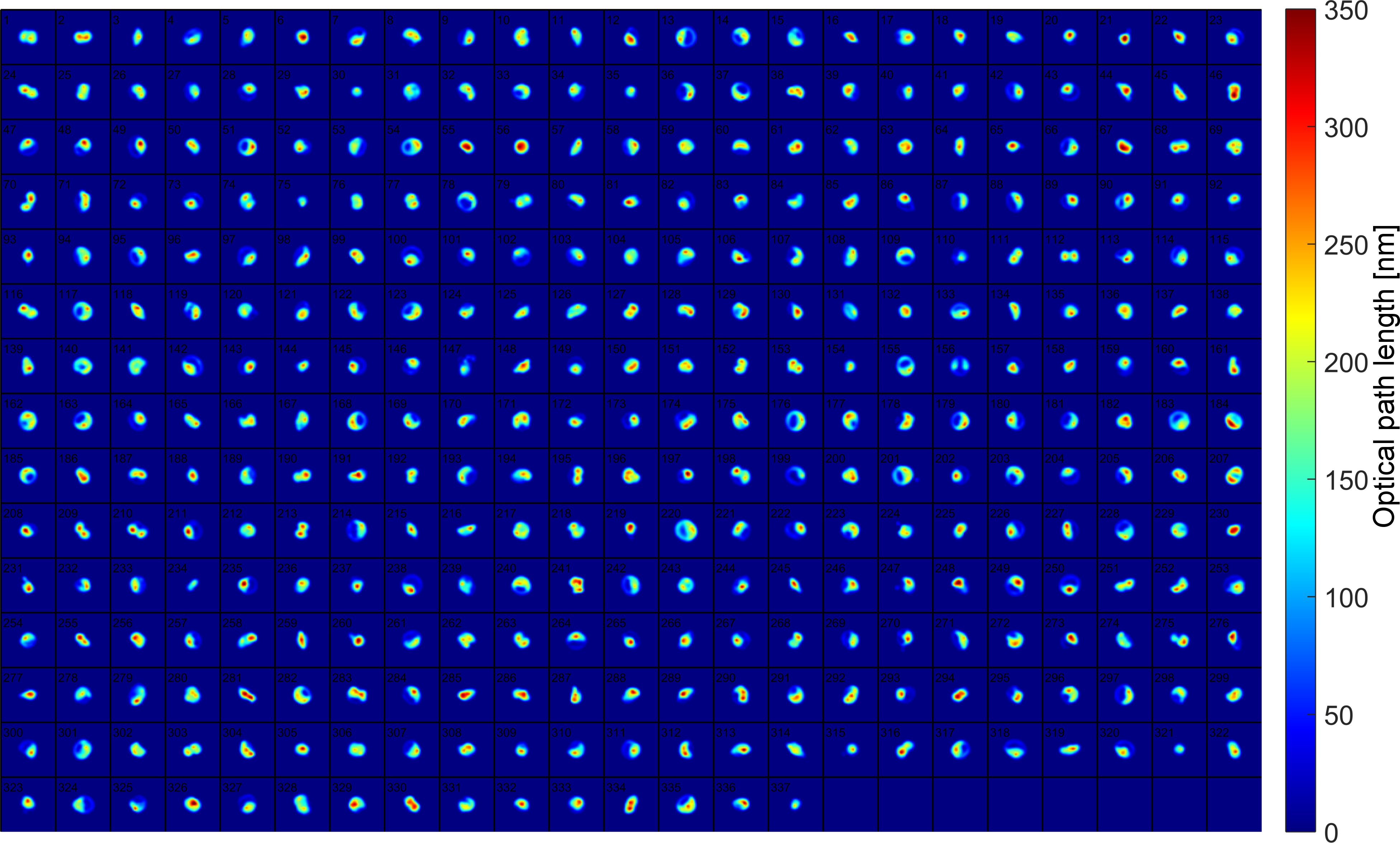

Supplement: S4 Fig — RBCs infected with P.falciparum in schizont stage, N = 337 (square tile = 20μm x 20μm). (TIF) [file pone.0163045.s004.tif]
